# Linear scalability of virus production in the integrity^® ^iCELLis^® ^single-use fixed-bed bioreactors from bench to industrial scale

**DOI:** 10.1186/1753-6561-7-S6-P60

**Published:** 2013-12-04

**Authors:** Shane Knowles, Jean-Christophe Drugmand, Nicolas Vertommen, Jose Castillo

**Affiliations:** 1 ATMI LifeSciences, Rue de Ransbeek 310, Brussels, Belgium, 1120

## Introduction

In order to maximize cell growth within a compact space and retain cells for easy medium exchange, the iCELLis bioreactors from ATMI LifeSciences contain macro-carriers trapped in a fixed-bed, creating a 3-D matrix within which cells adhere and replicate. These bioreactors also enable precise temperature, pH and dissolved oxygen control which cannot be done in 2-D cultures.

The iCELLis technology can be used at small and large scales with straightforward process scale-up, easy single-use operations and minimal space requirement.

Here we present a summary of adherent cell process development in iCELLis bioreactors, including:

• HEK 293 cell expansion for production of adenovirus

• MVA virus production in CEF cells

• Bovine Herpes Virus production in MDBK cells

• Recombinant Adeno-Associated Virus in A549 cells

• Adenovirus production in A549 cells

• Influenza virus production in Vero cells

• Paramyxovirus production in Vero cells

• Undisclosed lytic virus in Vero cells

## Transfer and scale-up of a HEK293 cell culture process for production of adenovirus

Small Scale Development

An existing process using HEK293 cells for the production of adenovirus was first transferred from multi-tray systems to an iCELLis nano bioreactor (0.53 m^2^, 40 ml of fixed-bed) by keeping equivalent cell culture parameters:

• Temperature, pH, DO (% saturation with air)

• Multiplicity of infection (pfu/cell)

• Time of infection

• Cell seeding density (cells/cm^2 ^and cells/mL)

• Culture duration

Additional experiments were performed with lower cell densities at inoculation in order to reduce the number of pre-culture steps at large scale. The following parameters were also optimized for cell growth and virus productivity:

• Compaction of carriers inside the fixed-bed (96 g/L or 144 g/L)

• Linear velocity of medium through the fixed-bed (cm/s).

• Fixed-bed height (2,4 or 10 cm)

## Industrial scale-up

The scale-up of iCELLis technology is similar to that of chromatography columns. The difference in fixed bed geometry from small to large scale is that the cross-sectional area increases, while the fixed-bed (FB) height remains constant. Therefore, cell seeding, nutrient and oxygen delivery throughout the fixed bed are comparable at small and large scale.

**Figure 1:**
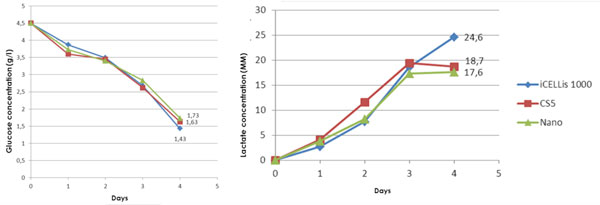
**Comparability of Glucose (Top Panel) and Lactate (Bottom Panel) Profiles of HEK293 culture in iCELLis 133 m^2 ^(Blue), iCELLis nano 1.06 m^2 ^(Green) and 5-tray Cell Factory (Red)**.

After determining optimal parameters at small scale, HEK293 cell culture batches were performed in duplicate with small and large scale bioreactors. Inoculation density, medium volume ratios, culture duration, pH, DO and temperature set points were kept identical. Consistent cell densities of 2.7 to 3.8 cells/cm2 were achieved in multiple experiments at both small and large scale. Analysis of glucose and lactate (Figure 1) at both scales in comparison to a 5-tray Cell Factory control indicated that cell metabolism was comparable between small and large scale iCELLis bioreactors and the standard 2D process.

## Additional Virus Production Process Development

Results of experiments performed for production of several viruses in various cell lines at various bioreactor scales are shown in Table 1. Bench scale bioreactors were used for each process to determine what conditions and feeding strategies sustained the highest growth rates and cell densities.

**Table 1:** Summary of results of virus production processes tested in various cell lines in iCELLis bioreactors (or predecessors).

Cells	Virus	Bioreactor	Surface Area (m^2^)	Average Cell Density at TOI (cells/cm^2^)	Specific Virus Productivity		Total Virus	
**CEF**	Modified Vaccina Ankara	Artefix	0.07	3.9E+05	3.0E+06	pfu/cm^2^	2.1E+09	pfu

**MDBK**	Bovine Herpes Virus	iCELLis nano	4	1.2E+05	2.2E+07	pfu/cm^2^	8.7E+11	pfu
		iCELLis pilot	20	1.4E+05	1.7E+07	pfu/cm^2^	3.4E+12	pfu
		iCELLis 500	66	3.3E+05	3.3E+07	pfu/cm^2^	2.2E+13	pfu

**A549**	rAAV	iCeLLis nano	0.53	6.0E+04	5.3E+08	vg/cm^2^	2.8E+12	vg
	Adenovirus	iCELLis nano	2.67	2.3E+05	1.1E+10	TCID50/cm^2^	3.0E+14	TCID50

**Vero**	Influenza	iCELLis nano	4	1.0E+05	3.8E+06	TCID50/cm^2^	1.5E+11	TCID50
		iCELLis pilot	20	7.5E+04	2.5E+06	TCID50/cm^2^	5.0E+11	TCID50
	Paramyxovirus	iCELLis nano	2.67	2.7E+05	6.4E+05	TCID50/cm^2^	1.7E+10	pfu

**Vero**	Undisclosed Lytic Virus	iCELLis pilot	40	1.5E+05	Confidential		Confidential	
		iCELLis 500	133	1.5E+05				
		iCELLis 1000	660	2.3E+05				

Bench scale bioreactors were used for each process to determine what conditions and feeding strategies sustained the highest growth rates and cell densities.

For chicken embryonic fibroblasts (CEF) and production of Modified Vaccinia Ankara (MVA), a prototype "Artefix" bioreactor (the predecessor of iCELLis) with a 0.07 m^2 ^fixed-bed surface area was tested.

Intermediate "pilot" scale prototype iCELLis bioreactors with surface areas of 20 or 40 m^2 ^were used to test Vero and MDBK cell processes.

The Vero cell process was scaled up to a 660 m^2 ^bioreactor. In this case, cells were inoculated at only 3200 cells/cm^2 ^using two 40-tray Cell Factories (2.5 m^2 ^each), equivalent to fifteen roller bottles (1700 cm^2 ^each). With such a low seeding density the seed train required for inoculation is simplified extensively compared to standard 2D cell culture processes. The Vero cell density reached 2.3 × 10^5 ^cells/cm^2 ^for a total biomass of 1.5 × 10^12 ^cells in 11 days. A complete medium exchange was then performed, followed by virus infection. Continuous perfusion of medium was used during the production phase. While the virus type and productivity data is confidential, the results indicated that virus output was equivalent or better than expected based on the standard 2D process.

## Conclusions

This summary of experiments demonstrates that the fixed-bed design of the iCELLis bioreactor enables high cell densities to be achieved and maintained in both small and large bioreactor volumes. Different processes have been easily scaled up by keeping cell culture conditions and process parameters identical to the standard 2-D cell culture process.

The iCELLis bioreactor can be inoculated at a very low cell density, leading to a dramatic simplification of seed train operations and a significant reduction of development timelines.

In conclusion, large biomass amplification and excellent virus productivities, combined with the advantages of a fully closed disposable system with low shear stress, make the iCELLis fixed-bed bioreactor a simple and straightforward solution for industrial production of viruses.

